# An untypeable enterotoxigenic *Escherichia coli* represents one of the dominant types causing human disease

**DOI:** 10.1099/mgen.0.000121

**Published:** 2017-07-03

**Authors:** Atsushi Iguchi, Astrid von Mentzer, Taisei Kikuchi, Nicholas R. Thomson

**Affiliations:** ^1^​ University of Miyazaki, Miyazaki, Japan; ^2^​ University of Gothenburg, Gothenburg, Sweden; ^3^​ Wellcome Trust Sanger Institute, Hinxton, UK

**Keywords:** enterotoxigenic *Escherichia coli*, O-antigen biosynthesis gene cluster, O genotype, PCR

## Abstract

Enterotoxigenic *Escherichia coli* (ETEC) is a major cause of diarrhoea in children below 5 years of age in endemic areas, and is a primary cause of diarrhoea in travellers visiting developing countries. Epidemiological analysis of *E. coli* pathovars is traditionally carried out based on the results of serotyping. However, genomic analysis of a global ETEC collection of 362 isolates taken from patients revealed nine novel O-antigen biosynthesis gene clusters that were previously unrecognized, and have collectively been called unclassified. When put in the context of all isolates sequenced, one of the novel O-genotypes, OgN5, was found to be the second most common ETEC O-genotype causing disease, after O6, in a globally representative ETEC collection. It’s also clear that ETEC OgN5 isolates have spread globally. These novel O-genotypes have now been included in our comprehensive O-genotyping scheme, and can be detected using a PCR-based and an *in silico* typing method. This will assist in epidemiological studies, as well as in ETEC vaccine development.

## Abbreviations

CF, colonization factor; ETEC, enterotoxigenic Escherichia coli; LT, heat-labile toxin; O-AGC, O-antigen biosynthesis gene cluster; OgUT, O-genotype untypeable; ST, heat-stable toxin; STEC, Shiga toxin-producing Escherichia coli.

## Data Summary

1. Assembled draft genomes for 55 enterotoxigenic *Escherichia coli* isolates were deposited in GenBank/EMBL/DDBJ under the accession numbers shown in Table S1 (available with the online Supplementary Material).

2. Sequences of nine annotated O-antigen biosynthesis gene clusters and three *wzy* genes from positive control strains (OT-37 for OgN3, EHOUT43 for OgN5 and EH-OSB16 for OSB16) were deposited in GenBank/EMBL/DDBJ under the accession numbers LC177546–LC177554 and LC223608–LC223610, respectively.

## Impact Statement

We identified nine novel O-genotypes (OgN) in a global collection of enterotoxigenic *Escherichia coli* (ETEC) isolates. The novel O-genotype OgN5 was found to be the second most common ETEC O-genotype globally, with no prior information regarding the contribution to the burden of disease. To gain more information about trends in ETEC OgN epidemiology, further studies of global OgN isolates are needed. The PCR method described in this study and an *in silico* typing method may help the surveillance and monitoring of the OgN groups.

## Introduction

The first diarrhoeal illness that infants often experience in endemic areas is caused by enterotoxigenic *Escherichia coli* (ETEC) [1]. In 2010, annual mortality from illness due to ETEC was estimated at 157 000 deaths (9 % of all deaths attributed to diarrhoea) and approximately 1 % of all deaths in children 28 days to 5 years of age [2]. Additionally, ETEC is a primary cause of diarrhoea in travellers visiting developing countries. In our previous study, we sequenced 362 globally representative ETEC isolates collected between 1980 and 2011 from 20 countries, including isolates from children and adults in endemic areas, as well as from travellers visiting such areas [3]. The majority of the isolates were collected from patients with diarrhoea. Genome-wide analysis showed that, contrary to previous understanding, there are long-term stable associations of ETEC lineages with specific virulence factors, such as plasmid-encoded heat-labile toxin (LT) and/or heat-stable toxin (ST; including two subtypes, STh and STp), and colonization factors (CFs), and that these lineages are globally distributed.

O-serogrouping remains the gold standard for the subtyping of *E. coli* isolates, especially pathogenic *E. coli*, for taxonomical and epidemiological studies. Most of what we know about *E. coli* prevalence, outbreaks and surveillance is described in terms of the O-serogroup. Recently, sequence analyses show that phenotypic O-serogroup diversification can be correlated with differences in the gene content and genetic diversity of the O-antigen biosynthesis gene cluster (O-AGC) located on the chromosome [4]. In particular, sequences from O-antigen processing genes, such as *wzx* (encoding the O-antigen flippase), *wzy* (encoding the O-antigen polymerase), and the *wzm* and *wzt* genes (encoding components of the ABC transporter pathway) located on the O-AGCs are highly variable in sequence, and can be used as gene markers for the identification of O-serogroups via molecular approaches [4]. By applying an *in silico* blast analysis using a *wzx*/*wzy* and *wzm*/*wzt* sequence set extracted from O1 to O187 O-AGCs, we subtyped our 362 global ETEC isolates [3] into 48 O-genotypes, of which the top 6 were O6 (*n*=38), O25 (*n*=24), O27 (*n*=18), O114 (*n*=17), O115 and O159 (*n*=16, each). In addition to the ETEC isolates classified into 48 known O-genotypes, 55 isolates carried *wzx*/*wzy* genes that showed <50 % or no sequence identity to the previously defined sequences. These ETEC isolates were categorized as O-genotype untypeable (OgUT), but carried novel O-AGCs.

In this study, we focused on characterizing the OgUT ETEC isolates detected in our previous study [3]. Such ETEC isolates may not be recognized by public-health surveillance, because they cannot be assigned to any known O-genotype and/or O-serogroup. To determine the relative contribution of OgUT ETEC isolates, we defined the novel O-genotypes of these ETEC isolates and estimated their overall contribution to disease by screening our global collection of ETEC.

## Methods

### Source sequences

ETEC O-AGC sequences were obtained from draft genomes used in a previous study [3]. The *wzx* and *wzy* sequences were extracted from each O-AGC. The known *wzx*/*wzy* sequences from typical *E. coli* O-serogroups (171 groups, O1–O187 except for *wzm*/*wzt*-type groups) [4], *Shigella* O-serogroups (total 34 types; 13 from *Shigella dysenteriae*, 18 from *Shigella boydii*, 2 from *Shigella flexnerii* and a single type of *Shigella sonnei*) [5], *E. coli* OX serogroups (11 groups: OX6, OX9, OX10, OX13, OX18, OX19, OX21, OX25, OX28, OX38, OX43) [6] and 6 recently defined novel O genotypes from Shiga toxin-producing *E. coli* (OgN1, OgN8, OgN9, OgN10, OgN12, OgN31) [7] were used (except for *wzy* genes of OX25 and *S. dysenteriae* type 6, which are not found in the O-AGCs). A complete set of *fliC* sequences [8] was also used for molecular-based H-typing.

### Sequence comparisons

Phylogenetic trees of *wzx* and *wzy* were constructed by using the neighbour-joining algorithm using mega5 software [9], following multiple alignments of nucleotide sequences by the clustal W program [10].

### PCR

OgN-specific PCR primers targeting OgN3, OgN5 and OSB16 were designed based on each alignment of *wzy* sequences. PCR was performed as follows: the 30 µl reaction mixture contained 2 µl genomic DNA, 6 µl 5× Kapa *Taq* buffer, dNTP mix (final concentration 0.3 mM each), MgCl_2_ (final concentration 2.5 mM), primers (final concentration 0.5 µM each) and 0.8 U Kapa *Taq* DNA polymerase (Kapa Biosystems). The thermocycling conditions were: 25 cycles of 94 °C for 30 s, 58 °C for 30 s and 72 °C for 1 min. The PCR products were visualized following agarose gel (1.5 %) electrophoresis in 0.5x TBE (25 mM Tris borate, 0.5 mM EDTA) and staining with ethidium bromide (1 mg/ml). Three strains (OT-37 for OgN3, EHOUT43 for OgN5 and EH-OSB16 for OSB16) were used as positive-control strains for PCR.

## Results

**Fig. 1.**
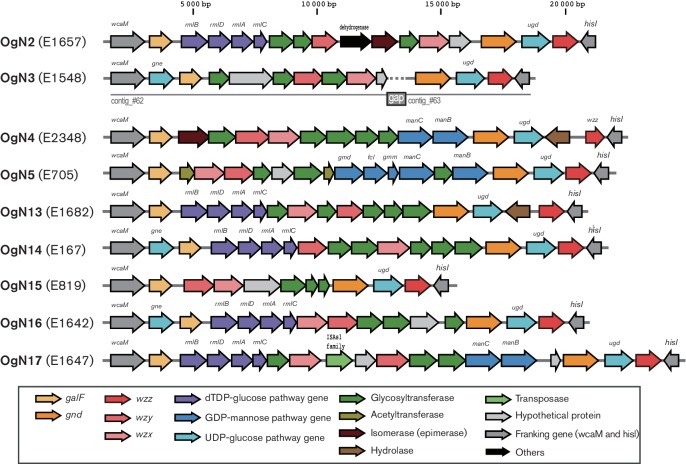
Nine novel O-antigen biosynthesis gene clysters from the ETEC collection.

**Fig. 2.**
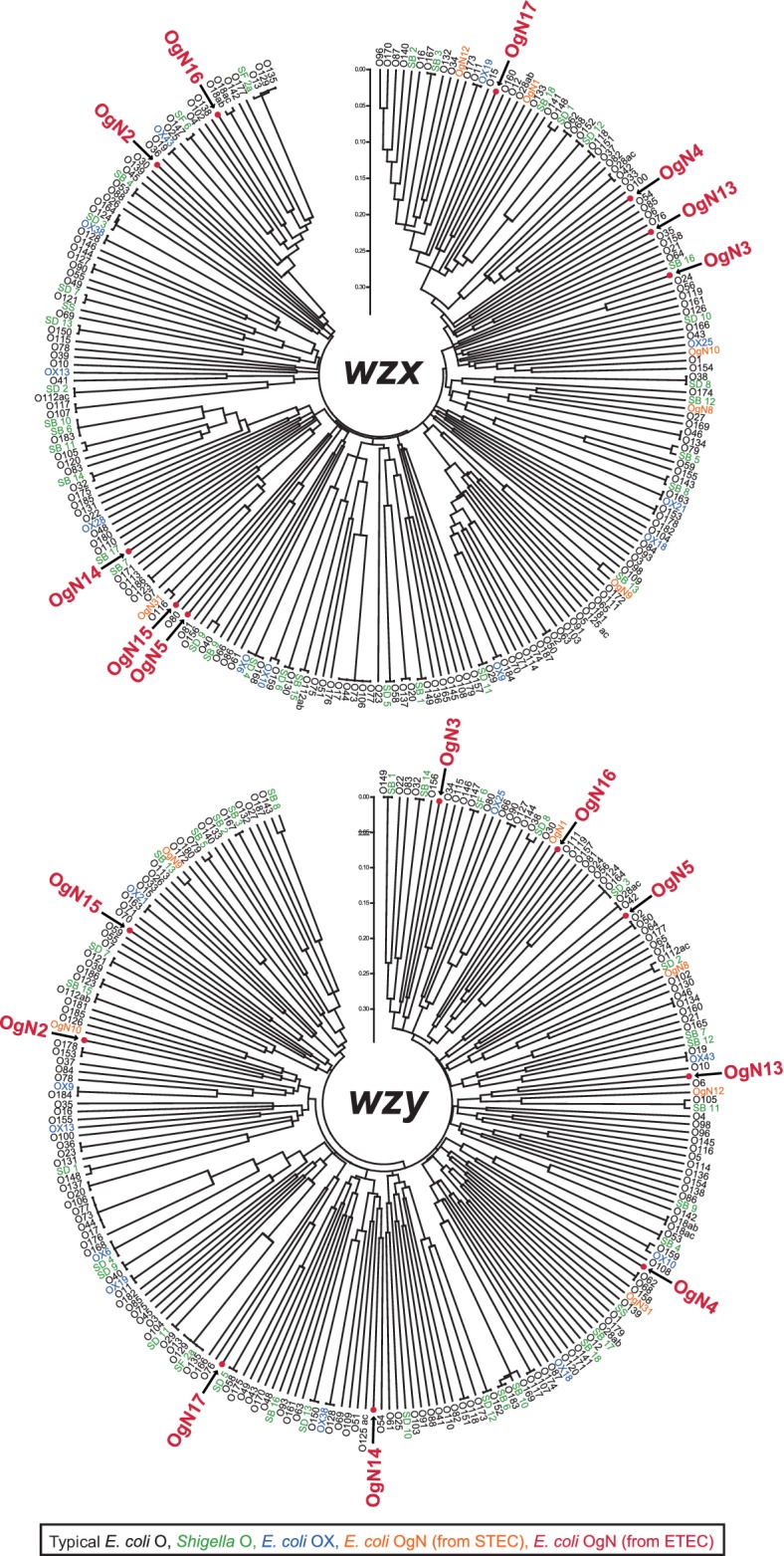
Phylogenetic analysis of *wzx* and *wzy* homologues from nine novel O-AGCs of ETEC and known *E. coli* and *Shigella* O-serogroups. STEC, Shiga toxin-producing *E. coli*.

**Fig. 3.**
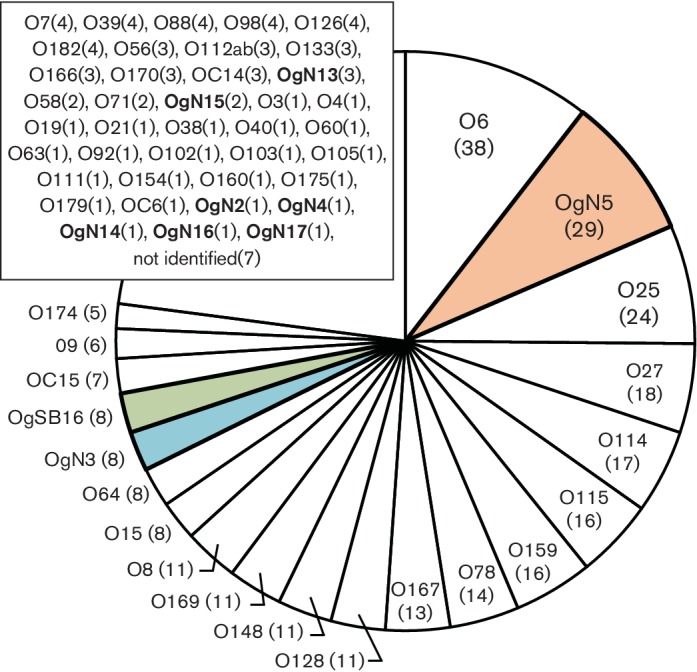
Molecular-based O-serogroup distribution of a global collection of 362 ETEC isolates. The numbers in parentheses indicate the number of isolates.

Comparative analyses revealed that chromosomal O-AGC sequences flanked by gene clusters of the colanic acid biosynthesis (*wca*) (upstream) and the histidine biosynthesis (*his*) (downstream) extracted from OgUT ETEC genomes contained nine novel O-AGCs, OgN2, OgN3, OgN4, OgN5, OgN13, OgN14, OgN15, OgN16 and OgN17 (Fig. 1), which all carried unique *wzx*/*wzy* genes, and showed <70 % nucleotide sequence identities among those from known *E. coli* O-AGCs (Fig. 2). One of the O-AGCs was similar (the same gene construction and ≥97 % sequence identity) to that of *S. boydii* type 16 (OSB16) (Fig. 2). Among the novel O-AGC sequences identified in our ETEC collection, it was apparent that the novel OgN5 was carried by more than half of ETEC OgUT isolates (29/55), followed in frequency by OgN3 and OSB16, 8 isolates each (Fig. 3). A summary of the epidemiological and genotypic data for the novel and SB16 O-genotype isolates is shown in Table S1. Sequences of *wzx* and *wzy* were highly conserved within each O-genotype (99.8–100 % sequence identity). ETEC OgN5 strains were isolated in five countries, Guatemala (*n*=12), Argentina (*n*=5), Egypt (*n*=4), Mexico (*n*=4) and Indonesia (*n*=4), between 1989 and 2003. All ETEC OgN5 isolates carried the ST-encoding gene, except for a single isolate (E1542) that carried the LT-encoding gene, and all OgN5 isolates were confirmed negative for all known CFs by dot-blot and PCR-based analyses in our previous study [3]. The *fliC*-based H-typing showed that the OgN5 isolates were classified into eight H-types: H5, H9, H10, H16, H18, H19, H32 and H39 (see Table S1). Phylogenetic analysis showed OgN5 isolates were spread across phylogenetically distinct lineages where most/some isolates grouped based on their H-types (see Fig. S1, Table S1). The major groups were OgN5 : H10 and OgN5 : H16 (*n*=6, each) belonging to a phylogenetic lineage L14 [3] of *E. coli* phylogroup A and a lineage L22 (designed in this study) of phylogroup B, respectively (see Fig. S1). Additionally, OgN5 : H18 (L11, *n*=1), OgN5 : H9 (L14, *n*=1), OgN5 : H32 (L15, *n*=4), OgN5 : H19 (L15, *n*=1), OgN5 : H34 (L16, *n*=4), OgN5 : H16 (L23, *n*=3) and OgN5 : H5 (L24, *n*=3) were observed. No significant association between lineages and geographical origins was observed (see Table S1).

Six of eight OgN3 strains carried the *fliC* of H45, and were confirmed positive for STh and two CFs, CFA/I (a fimbria) and CS21 (a type IV pilus) (see Table S1). All OgN3 : H45 isolates were phylogenetically grouped into L6 of phylogroup A, and originated from Central and South America, including Mexico, Guatemala and Argentina (see Fig. S1, Table S1). Six of eight OSB16 strains carried the *fliC* of H32, and were confirmed positive for LT, LT+STh or LT+STp, and negative for all known CFs (see Table S1). All OSB16 : H32 and OSB16 : H2 isolates were phylogenetically grouped into L13 of phylogroup A, and originated from Guatemala and Argentina (see Fig. S1, Table S1). Additionally, three OgN13, two OgN15 and single OgN2, OgN4, OgN14, OgN16 and OgN17 strains were observed in our ETEC collection (see Table S1).

**Fig. 4.**
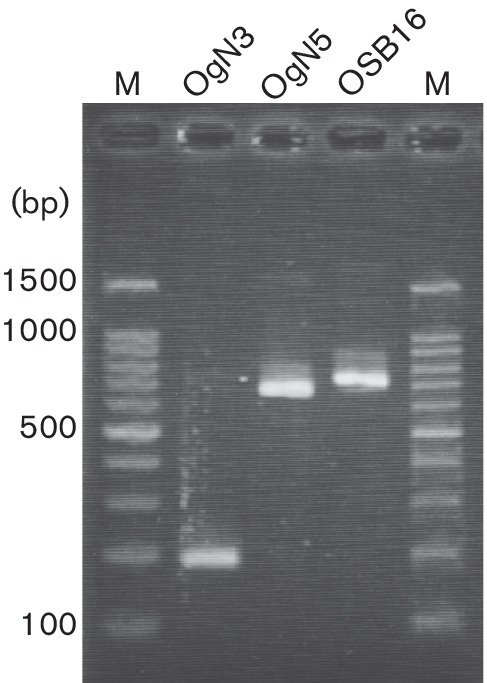
Gel image of PCR products obtained using the three primer pairs and positive control strains [OgN3, OT-37 (189 bp); OgN5, EHOUT43 (650 bp); OSB16, EH-OSB16 (717 bp)]. M, Marker.

To aid in the identification of the predominant novel ETEC O-AGCs, we designed specific PCR primers for identifying OgN5, OgN3 and OSB16 targeting unique sequences on *wzy* genes (Table 1, Fig. 4), and their specificities were confirmed by using positive-control strains and all O-serogroup reference strains (O1–O188).

**Table 1.** PCR primers for identifying OgN3, OgN5 and OSB16

O genotype	Primer name	Sequence (5′→3′)	Target gene	Size (bp)
OgN3	OgN3_PCR_F	GCTTGGCATCGTTGGGGATA	*wzy*	189
	OgN3_PCR_R	TGCTACCAATCAGGCCGCTA		
OgN5	OgN5_PCR_F	GGTTTAAGCGACCCGTATCG	*wzy*	650
	OgN5_PCR_R	CCAATTCCAGCCAGTGATGAG		
OSB16	OgSB16_PCR_F	AACCGCAGTGGAAACTGCA	*wzy*	717
	OgSB16_PCR_R	AATTCCACATCAATCCACGGA		

## Discussion

The results of the current study encapsulate problems with using a limited number of phenotypic markers for typing and tracking pathogenic bacteria of importance to human or animal health. It also highlights how, with whole-genome sequencing, there are still major discoveries to be made in identifying the isolates/lineages or types that are responsible for a significant burden of reported disease and yet untypeable by traditional methods. Since the O-serogroup is still used as a general marker for the subtyping of *E. coli* isolates, epidemiologically there can be an unawareness of the presence, emergence or spread of O-serogroup untypeable pathogenic *E. coli* groups. In this study, we used extracted O-AGC sequences from 362 ETEC genomes and by comparative genomics revealed nine novel O-genotypes that were previously unrecognized by the standard methods used for serotype detection and surveillance.

In recent surveillance studies, O6 has been shown to be the most common ETEC O-serogroup (mostly O6 : H16 serotype) associated with diarrhoeal patients in Egypt [11], Bolivia [12], Bangladesh [13], China [14] and Japan [15]. The O6-serogroup is mainly found in isolates with the CF profile CS1+CS2 and CS1+CS3, which are three of the most prevalent CFs identified in clinical isolates [16]. In our ETEC collection, the most prevalent O-serogroup was O6. O25 is also a well-known ETEC O-serogroup frequently isolated from ETEC patients, such as in Bosnia and Herzegovina [17], Bangladesh [13], China [14] and Japan [15]. Interestingly, in our representative ETEC collection, the novel O-genotype OgN5 was the second most common ETEC after O6 and before O25. Moreover, ETEC OgN5 isolates included those collected from children and adults in endemic regions such as Argentina, Egypt, Guatemala and Indonesia, as well as from travellers to Guatemala and Mexico, between 1989 and 2003. These data indicate that OgN5-positive ETEC isolates have spread both globally and over time.

Novel O-genotype ETEC isolates were found in several distinct phylogenetic lineages, and sequences of *wzx*/*wzy* from O-AGCs were highly conserved within each O-genotype, indicating that these O-AGCs have been spread across this species by horizontal gene transfer. Phylogenetic analysis in our previous study [3] showed that most ETEC isolates clustered based on their O-serogroups, such as O6 isolates belonging to L1+L2 (37/38), O25 belonging to L4 (22/24), O27 belonging to L9+L17 (18/18), O114 belonging to L4+L10 (14/17) and O115 belonging to L5 (16/16). In this study, ETEC OgN3 and OSB16 were mostly clustered in L16 and L13, respectively. However, OgN5 isolates were found in several distinct lineages, suggesting frequent exchanges of the O-AGC occur between lineages. Horizontal gene transfer-promoting sequences, such as transposase genes and repeat sequences, were not found in and around the OgN5 O-AGC.

Recently, we have been able to access whole-genome sequencing data due to the appearance of next-generation sequence technologies. A publicly available *in silico*-based serotyping web tool, SerotypeFinder, of the Center for Genomic Epidemiology (https://cge.cbs.dtu.dk/services/SerotypeFinder) [8], which has a database consisting of *E. coli* O-antigen associated genes (*wzx, wzy, wzm* and *wzt*) from O1 to O187 and H-antigen associated genes (*fliC* and its homologues) from H1 to H56 can be used to estimate *E. coli* O : H serotypes of sequenced isolates. If it is possible to organize and update the database with new *wzx* and *wzy* sequences, it becomes possible to meet the requirements for broader O-genotypes, including OgN5, OgN3 and OSB16 in the *in silico* typing.

In conclusion, by using detailed whole genome sequence-based analyses, it is clear that there is a hidden diversity of ETEC O-genotypes for which there is no prior information regarding their contribution to the burden of disease. It should be noted that even from our global collection, ETEC isolates from Africa are underrepresented. Hence, it is possible and perhaps likely that additional novel ETEC O-types and their associated lineages will continue to be discovered. These novel O-genotypes identified in this study have now been included in our comprehensive O-genotyping scheme and can be detected using the PCR-based method described in this study and by the *in silico* typing method.

## Data bibliography

1. Bastin DA, Reeves PR. Sequence and analysis of the O antigen gene (*rfb*) cluster of *Escherichia coli* O111. *Gene* 1995;16:17–23. GenBank/EMBL/DDBJ accession no.: AF078736.
2. Beutin L, Kong Q, Feng L, Wang Q, Krause G *et al.*Development of PCR assays targeting the genes involved in synthesis and assembly of the new *Escherichia coli* O 174 and O 177 O antigens. *J Clin Microbiol* 2005;43:5143–5149. GenBank/EMBL/DDBJ accession no.: DQ008593.
3. Beutin L, Tao J, Feng L, Krause G, Zimmermann S *et al.* Sequence analysis of the *Escherichia coli* O15 antigen gene cluster and development of a PCR assay for rapid detection of intestinal and extraintestinal pathogenic *E. coli* O15 strains. *J Clin Microbiol* 2005;43:703–710. GenBank/EMBL/DDBJ accession no.: AY647261.
4. Cheng J, Wang Q, Wang W, Wang Y, Wang L *et al.* Characterization of *E. coli* O24 and O56 O antigen gene clusters reveals a complex evolutionary history of the O24 gene cluster. *Curr Microbiol* 2006;53:470–476. GenBank/EMBL/DDBJ accession nos: DQ220292, DQ220293.
5. Cheng J, Liu B, Bastin DA, Han W, Wang L *et al.* Genetic characterization of the *Escherichia coli* O66 antigen and functional identification of its wzy gene. *J Microbiol* 2007;45:69–74. GenBank/EMBL/DDBJ accession no.: DQ069297.
6. Cunneen MM, Reeves PR. The *Yersinia kristensenii* O11 O-antigen gene cluster was acquired by lateral gene transfer and incorporated at a novel chromosomal locus. *Mol Biol Evol* 2007;24:1355–1365. GenBank/EMBL/DDBJ accession no.: DQ180602.
7. D'Souza JM, Samuel GN, Reeves PR. Evolutionary origins and sequence of the *Escherichia coli* O4 O-antigen gene cluster. *FEMS Microbiol Lett* 2005;244:27–32. GenBank/EMBL/DDBJ accession no.: AY568960.
8. D'Souza JM, Wang L, Reeves P. Sequence of the *Escherichia coli* O26 O antigen gene cluster and identification of O26 specific genes. *Gene* 2002;297:123–127. GenBank/EMBL/DDBJ accession no.: AF529080.
9. DebRoy C, Fratamico PM, Roberts E, Davis MA, Liu Y. Development of PCR assays targeting genes in O-antigen gene clusters for detection and identification of *Escherichia coli* O45 and O55 serogroups. *Appl Environ Microbiol* 2005;71:4919–4924. GenBank/EMBL/DDBJ accession no.: AY771223.
10. DebRoy C, Fratamico PM, Yan X, Baranzoni G, Liu Y, *et al.* Comparison of O-antigen gene clusters of all O-serogroups of *Escherichia coli* and proposal for adopting a new nomenclature for O-typing. *PLoS One* 2016;11:e0147434. GenBank/EMBL/DDBJ accession nos: KJ778794, KJ778795, KJ778792, KP710591, KJ710507, KP868751, KJ739596, KP710594, KT207929, KJ739599, KP835691.
11. Feng L, Senchenkova SN, Yang J, Shashkov AS, Tao J, *et al.* Structural and genetic characterization of the *Shigella boydii* type 13 O antigen. J Bacteriol 2004;186:383–392. GenBank/EMBL/DDBJ accession no.: AY369140.
12. Feng L, Tao J, Guo H, Xu J, Li Y *et al.* Structure of the *Shigella dysenteriae* 7 O antigen gene cluster and identification of its antigen specific genes. *Microb Pathog* 2004;36:109–115. GenBank/EMBL/DDBJ accession no.: AY380835.
13. Feng L, Senchenkova SN, Yang J, Shashkov AS, Tao J *et al.* Synthesis of the heteropolysaccharide O antigen of *Escherichia coli* O52 requires an ABC transporter: structural and genetic evidence. *J Bacteriol* 2004;186:4510–4519. GenBank/EMBL/DDBJ accession no.: AY528413.
14. Feng L, Wang W, Tao J, Guo H, Krause G *et al.* Identification of *Escherichia coli* O114 O-antigen gene cluster and development of an O114 serogroup-specific PCR assay. *J Clin Microbiol* 2004;42:3799–3804. GenBank/EMBL/DDBJ accession no.: AY573377.
15. Feng L, Senchenkova SN, Wang W, Shashkov AS, Liu B *et al.* Structural and genetic characterization of the *Shigella boydii* type 18 O antigen. *Gene* 2005;355:79–86. GenBank/EMBL/DDBJ accession no.: AY948196.
16. Feng L, Senchenkova SN, Tao J, Shashkov AS, Liu B *et al.* Structural and genetic characterization of enterohemorrhagic *Escherichia coli* O145 O antigen and development of an O145 serogroup-specific PCR assay. *J Bacteriol* 2005;187:758-64. GenBank/EMBL/DDBJ accession no.: AY647260.
17. Feng L, Perepelov AV, Zhao G, Shevelev SD, Wang Q *et al.* Structural and genetic evidence that the *Escherichia coli* O148 O antigen is the precursor of the *Shigella dysenteriae* type 1 O antigen and identification of a glucosyltransferase gene. *Microbiology* 2007;153:139–147. GenBank/EMBL/DDBJ accession nos: DQ000313, DQ167407.
18. Fratamico PM, Briggs CE, Needle D, Chen CY, DebRoy, C. Sequence of the *Escherichia coli* O121 O-antigen gene cluster and detection of enterohemorrhagic *E. coli* O121 by PCR amplification of the wzx and wzy genes. *J Clin Microbiol* 2003;41:3379–3383. GenBank/EMBL/DDBJ accession no.: AY208937.
19. Fratamico PM, DebRoy C, Strobaugh TP Jr, Chen CY. DNA sequence of the *Escherichia coli* O103 O antigen gene cluster and detection of enterohemorrhagic *E. coli* O103 by PCR amplification of the *wzx* and *wzy* genes. *Can J Microbiol* 2005;51:515–522. GenBank/EMBL/DDBJ accession no.: AY532664.
20. Guo H, Feng L, Tao J, Zhang C, Wang L. Identification of *Escherichia coli* O172 O-antigen gene cluster and development of a serogroup-specific PCR assay. *J Appl Microbiol* 2004;97:181–190. GenBank/EMBL/DDBJ accession no.: AY545992.
21. Guo H, Yi W, Shao J, Lu Y, Zhang W, *et al.* Molecular analysis of the O-antigen gene cluster of *Escherichia coli* O86:B7 and characterization of the chain length determinant gene (*wzz*). *Appl Environ Microbiol* 2005;71:7995–8001. GenBank/EMBL/DDBJ accession no.: AY220982.
22. Guo H, Kong Q, Cheng J, Wang L, Feng L. Characterization of the *Escherichia coli* O59 and O155 O-antigen gene clusters: the atypical wzx genes are evolutionary related. *FEMS Microbiol Lett* 2005;15:153–161. GenBank/EMBL/DDBJ accession nos: AY654590, AY657020.
23. Han W, Liu B, Cao B, Beutin L, Krüger U *et al.* DNA microarray-based identification of serogroups and virulence gene patterns of *Escherichia coli* isolates associated with porcine postweaning diarrhea and edema disease. *Appl Environ Microbiol* 2007;73:4082–4088. GenBank/EMBL/DDBJ accession nos: DQ868764–DQ868766.
24. Hu B, Perepelov AV, Liu B, Shevelev SD, Guo D *et al.* Structural and genetic evidence for the close relationship between *Escherichia coli* O71 and *Salmonella enterica* O28 O-antigens. *FEMS Immunol Med Microbiol* 2010;59:161–169. GenBank/EMBL/DDBJ accession no.: GU445927.
25. Iguchi A, Iyoda S, Seto K, Ohnishi M, EHEC Study Group. Emergence of a novel Shiga toxin-producing *Escherichia coli* O serogroup cross-reacting with *Shigella boydii* type 10. *J Clin Microbiol* 2011;49:3678–3680. GenBank/EMBL/DDBJ accession no.: AB627352.
26. Iguchi A, Iyoda S, Kikuchi T, Ogura Y, Katsura K *et al.* A complete view of the genetic diversity of the *Escherichia coli* O-antigen biosynthesis gene cluster. *DNA Res* 2015;22:101–107. GenBank/EMBL/DDBJ accession nos: AB811596–AB811624, AB812020–AB812085, AB972413–AB972424.
27. Iguchi A, Iyoda S, Seto K, Nishii H, Ohnishi M *et al.* Six novel O genotypes from Shiga toxin-producing *Escherichia coli*. *Front Microbiol* 2016;7:765. GenBank/EMBL/DDBJ accession nos: LC125927–LC125932.
28. Kido N, Torgov VI, Sugiyama T, Uchiya K, Sugihara H *et al.* Expression of the O9 polysaccharide of *Escherichia coli*: sequencing of the *E. coli* O9 rfb gene cluster, characterization of mannosyl transferases, and evidence for an ATP-binding cassette transport system. *J Bacteriol* 1995;177:2178-2187. GenBank/EMBL/DDBJ accession no.: D43637.
29. Li D, Liu B, Chen M, Guo D, Guo X *et al.* A multiplex PCR method to detect 14 *Escherichia coli* serogroups associated with urinary tract infections. *J Microbiol Methods* 2010;82:71-77. GenBank/EMBL/DDBJ accession nos: GU299791, GU299792, GU299795.
30. Li X, Perepelov AV, Wang Q, Senchenkova SN, Liu B *et al.* Structural and genetic characterization of the O-antigen of *Escherichia coli* O161 containing a derivative of a higher acidic diamino sugar, legionaminic acid. *Carbohydr Res* 2010;345:1581–1587. GenBank/EMBL/DDBJ accession nos: GU220361, GU220362.
31. Li Y, Perepelov AV, Guo D, Shevelev SD, Senchenkova SN *et al.* Structural and genetic relationships of two pairs of closely related O-antigens of *Escherichia coli* and *Salmonella enterica*: *E. coli* O11/*S. enterica* O16 and *E. coli* O21/*S. enterica* O38. *FEMS Immunol Med Microbiol* 2011;61:258–268. GenBank/EMBL/DDBJ accession no.: HQ388393.
32. Liu B, Senchenkova SN, Feng L, Perepelov AV, Xu T *et al.* Structural and molecular characterization of *Shigella boydii* type 16 O antigen. *Gene* 2006;380:46–53. GenBank/EMBL/DDBJ accession no.: DQ371800.
33. Liu B, Knirel YA, Feng L, Perepelov AV, Senchenkova SN *et al.* Structure and genetics of *Shigella* O antigens. *FEMS Microbiol Rev* 2008;32:627–653. GenBank/EMBL/DDBJ accession nos: EU294162–EU294178, EU296402–EU296404, EU296406–EU296418, EU296421.
34. Liu B, Wu F, Li D, Beutin L, Chen M *et al.* Development of a serogroup-specific DNA microarray for identification of *Escherichia coli* strains associated with bovine septicemia and diarrhea. *Vet Microbiol* 2009;142:373-378. GenBank/EMBL/DDBJ accession nos: FJ940774, FJ940775, GQ499368.
35. Liu B, Perepelov AV, Li D, Senchenkova SN, Han Y *et al.* Structure of the O-antigen of *Salmonella* O66 and the genetic basis for similarity and differences between the closely related O-antigens of *Escherichia coli* O166 and *Salmonella* O66. *Microbiology* 2010;156:1642–1649. GenBank/EMBL/DDBJ accession no.: GU299794.
36. Liu Y, DebRoy C, Fratamico P. Sequencing and analysis of the *Escherichia coli* serogroup O117, O126, and O146 O-antigen gene clusters and development of PCR assays targeting serogroup O117-, O126-, and O146-specific DNA sequences. *Mol Cell Probes* 2007;21:295–302. GenBank/EMBL/DDBJ accession no.: DQ465247-DQ465249.
37. Marolda CL, Feldman MF, Valvano MA. Genetic organization of the O7-specific lipopolysaccharide biosynthesis cluster of *Escherichia coli* VW187 (O7:K1). *Microbiology* 1999;145:2485–2495. GenBank/EMBL/DDBJ accession no.: AF125322.
38. Morona R, Mavris M, Fallarino A, Manning PA. Characterization of the rfc region of *Shigella flexneri*. *J Bacteriol* 1994;176:733–747. GenBank/EMBL/DDBJ accession no.: X71970.
39. Paton AW, Paton JC. Molecular characterization of the locus encoding biosynthesis of the lipopolysaccharide O antigen of *Escherichia coli* serotype O113. *Infect Immun* 1999;67:5930–5937. GenBank/EMBL/DDBJ accession no.: AF172324.
40. Perelle S, Dilasser F, Grout J, Fach P. Identification of the O-antigen biosynthesis genes of *Escherichia coli* O91 and development of a O91 PCR serotyping test. *J Appl Microbiol* 2002;93:758–764. GenBank/EMBL/DDBJ accession no.: AY035396.
41. Perepelov AV, Li D, Liu B, Senchenkova SN, Guo D *et al.* Structural and genetic characterization of the closely related O-antigens of *Escherichia coli* O85 and *Salmonella enterica* O17. *Innate Immun* 2008;17:164–73. GenBank/EMBL/DDBJ accession no.: GU299798.
42. Perepelov AV, Li D, Liu B, Senchenkova SN, Guo D *et al.* Structural and genetic characterization of *Escherichia coli* O99 antigen. *FEMS Immunol Med Microbiol* 2009;57:80–87. GenBank/EMBL/DDBJ accession no.: FJ940773.
43. Perepelov AV, Ni Z, Wang Q, Shevelev SD, Senchenkova SN *et al.* Structure and gene cluster of the O-antigen of *Escherichia coli* O109; chemical and genetic evidences of the presence of L-RhaN3N derivatives in the O-antigens of *E. coli* O109 and O119. *FEMS Immunol* *Med Microbiol* 2010;61:47–53. GenBank/EMBL/DDBJ accession no.: HM485572.
44. Ren Y, Liu B, Cheng J, Liu F, Feng L *et al.* Characterization of *Escherichia coli* O3 and O21 O antigen gene clusters and development of serogroup-specific PCR assays. *J Microbiol Methods* 2008;75:329–334. GenBank/EMBL/DDBJ accession no.: EU694097, EU694097.
45. Senchenkova SN, Feng L, Yang J, Shashkov AS, Cheng J *et al.* Structural and genetic characterization of the *Shigella boydii* type 10 and type 6 O antigens. *J Bacteriol* 2005;187:2551-2554. GenBank/EMBL/DDBJ accession no.: AY693427.
46. Senchenkova SN, Feng L, Wang Q, Perepelov AV, Qin D *et al.* Structural and genetic characterization of *Shigella boydii* type 17 O antigen and confirmation of two new genes involved in the synthesis of glucolactilic acid. *Biochem Biophys Res Commun* 2006;349:289–295. GenBank/EMBL/DDBJ accession no.: DQ875941.
47. Shao J, Li M, Jia Q, Lu Y, Wang PG. Sequence of *Escherichia coli* O128 antigen biosynthesis cluster and functional identification of an alpha-1,2-fucosyltransferase. *FEBS Lett* 2003;553:99–103. GenBank/EMBL/DDBJ accession no.: AY217096.
48. Tao J, Feng L, Guo H, Li Y, Wang L. The O-antigen gene cluster of *Shigella boydii* O11 and functional identification of its *wzy* gene. *FEMS Microbiol Lett* 2004;234:125–132. GenBank/EMBL/DDBJ accession no.: AY529126.
49. Tao J, Wang L, Liu D, Li Y, Bastin DA *et al.* Molecular analysis of *Shigella boydii* O1 O-antigen gene cluster and its PCR typing. *Can J Microbiol* 2005;51:387–392. GenBank/EMBL/DDBJ accession no.: AY630255.
50. Wang L, Reeves PR. Organization of *Escherichia coli* O157 O antigen gene cluster and identification of its specific genes. *Infect Immun* 1998;66:3545–3551. GenBank/EMBL/DDBJ accession no.: AF061251.
51. Wang L, Qu W, Reeves PR. Sequence analysis of four *Shigella boydii* O-antigen loci: implication for *Escherichia coli* and *Shigella* relationships. *Infect Immun* 2001;69:6923–6930. GenBank/EMBL/DDBJ accession no.: AF402312–AF402315.
52. Wang L, Briggs CE, Rothemund D, Fratamico P, Luchansky JB *et al.* Sequence of the *E. coli* O104 antigen gene cluster and identification of O104 specific genes. *Gene* 2001;30:231–236. GenBank/EMBL/DDBJ accession no.: AF361371.
53. Wang L, Huskic S, Cisterne A, Rothemund D, Reeves PR. The O-antigen gene cluster of *Escherichia coli* O55:H7 and identification of a new UDP-GlcNAc C4 epimerase gene. *J Bacteriol* 2002;184:2620–2625. GenBank/EMBL/DDBJ accession no.: AF461121.
54. Wang L, Liu B, Kong Q, Steinrück H, Krause G *et al.* Molecular markers for detection of pathogenic *Escherichia coli* strains belonging to serogroups O 138 and O 139. *Vet Microbiol* 2005;111:181–90. GenBank/EMBL/DDBJ accession nos: DQ109551, DQ109552.
55. Wang Q, Perepelov AV, Feng L, Knirel YA, Li Y *et al.* Genetic and structural analyses of *Escherichia coli* O107 and O117 O-antigens. *FEMS Immunol Med Microbiol* 2009;55:47–54. GenBank/EMBL/DDBJ accession no.: EU694095.
56. Wang Q, Wang S, Beutin L, Cao B, Feng L *et al.* Development of a DNA microarray for detection and serotyping of enterotoxigenic *Escherichia coli*. *J Clin Microbiol* 2010;48:2066–2074. GenBank/EMBL/DDBJ accession nos: GU014554, GU014555.
57. Wang Q, Ruan X, Wei D, Hu Z, Wu L *et al.* Development of a serogroup-specific multiplex PCR assay to detect a set of *Escherichia coli* serogroups based on the identification of their O-antigen gene clusters. *Mol Cell Probes* 2010;24:286–290. GenBank/EMBL/DDBJ accession nos: GU068041, GU068044–GU068046.
58. Xu DQ, Cisar JO, Ambulos Jr N, Burr DH *et al.* Molecular cloning and characterization of genes for *Shigella sonnei* form I O polysaccharide: proposed biosynthetic pathway and stable expression in a live salmonella vaccine vector. *Infect Immun* 2002;70:4414–4423. GenBank/EMBL/DDBJ accession no.: AF294823.
59. Xu DQ, Cisar JO, Osorio M, Wai TT, Kopecko DJ. Core-linked LPS expression of *Shigella dysenteriae* serotype 1 O-antigen in live *Salmonella* Typhi vaccine vector Ty21a: preclinical evidence of immunogenicity and protection. *Vaccine* 2007;25:6167–6175. GenBank/EMBL/DDBJ accession no.: AY585348.

## Supplementary Data

121 Supplementary File 1

## Supplementary Data

58 Supplementary File 2
