# Supplementary material for: An untypeable enterotoxigenic Escherichia coli represents one of the dominant types causing human disease
Source: Microb Genom. 2017 Jul 3;3(9):e000121. doi: 10.1099/mgen.0.000121 (PMC5643014; doi:10.1099/mgen.0.000121)
Supplement: Supplementary File 1 [file mgen-3-121-s001.pdf]

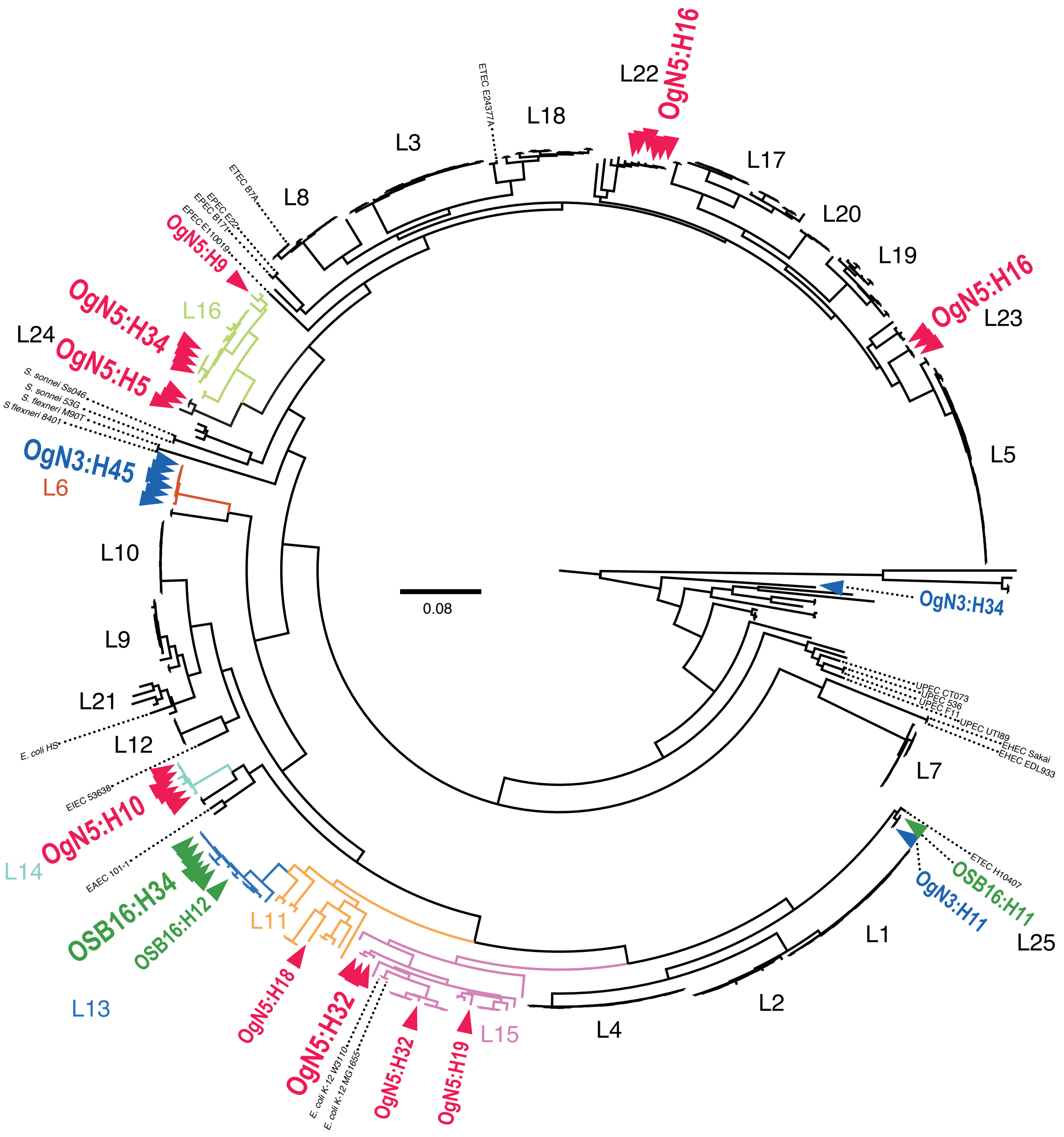

Fig. S1

Phylogenetic positions of OgN5, OgN3 and OSB16 isolates were overlaid onto the ETEC phylogenetic tree reconstructed based on the sequences obtaiend in our previous study (von Mentzer et al., 2014). Red arrowhead indicates OgN5 isolate, blue indicates OgN3 and green indicates OSB10. Lineages from L1 to L21 were assigned in our previous study (von Mentzer et al., 2014), and L22 to L25 were newly assigned in this study.

The tree was constructed based on the maximum common genome including 1,429 genes.
